# Mitral annular kinetics in mitral regurgitation

**DOI:** 10.1186/1532-429X-16-S1-P269

**Published:** 2014-01-16

**Authors:** Xiaoxia Zhang, Chun G Schiros, Mustafa I Ahmed, David S McGiffin, Steven G Lloyd, Louis J Dell'Italia, Thomas S Denney, Himanshu Gupta

**Affiliations:** 1 AU MRI Research Center, Auburn University, Auburn, Alabama, USA; 2 Electrical and Computer Engineering Department, Auburn University, Auburn, Alabama, USA; 3 Department of Medicine, Division of Cardiovascular Disease, University of Alabama at Birmingham, Birmingham, Alabama, USA; 4 VA Medical Center, Birmingham, Alabama, USA

## Background

Mitral annular (MA) kinetics during the cardiac cycle influences both systolic and diastolic function of the LV. Here we describe a novel cMRI approach to evaluate mitral annular kinetics and its relationship in mitral regurgitation post surgical repair.

## Methods

Population: Patients with degenerative mitral regurgitation (n = 35) with normal LV EF pre and 12 months post mitral valve repair. Normal volunteers (n = 51). Image Acquisition: All participants underwent MRI on a 1.5T scanner (GE Healthcare) optimized for cardiac application. An ECG-gated, breath-hold steady state free precision technique was used to obtain 2 chamber and 4 chamber views with 20 phase imaged in each cardiac cycle. Image Analysis: At end diastole (ED) and end systole (ES), anterior and inferior MA landmarks were manually marked on 2 chamber views. Septal, lateral and apex MA landmarks were marked on 4 chamber views. All landmarks were then automatically propagated to the remaining phases using custom software [1] and manually verified. In each phase, the MA was approximated by a flat plane fit to the 4 landmarks. The MA centroid and plane normal was computed in each plane. Differences in centroid position and plane normal angle were computed in each phase relative to ED.

## Results

**Figure 1:**
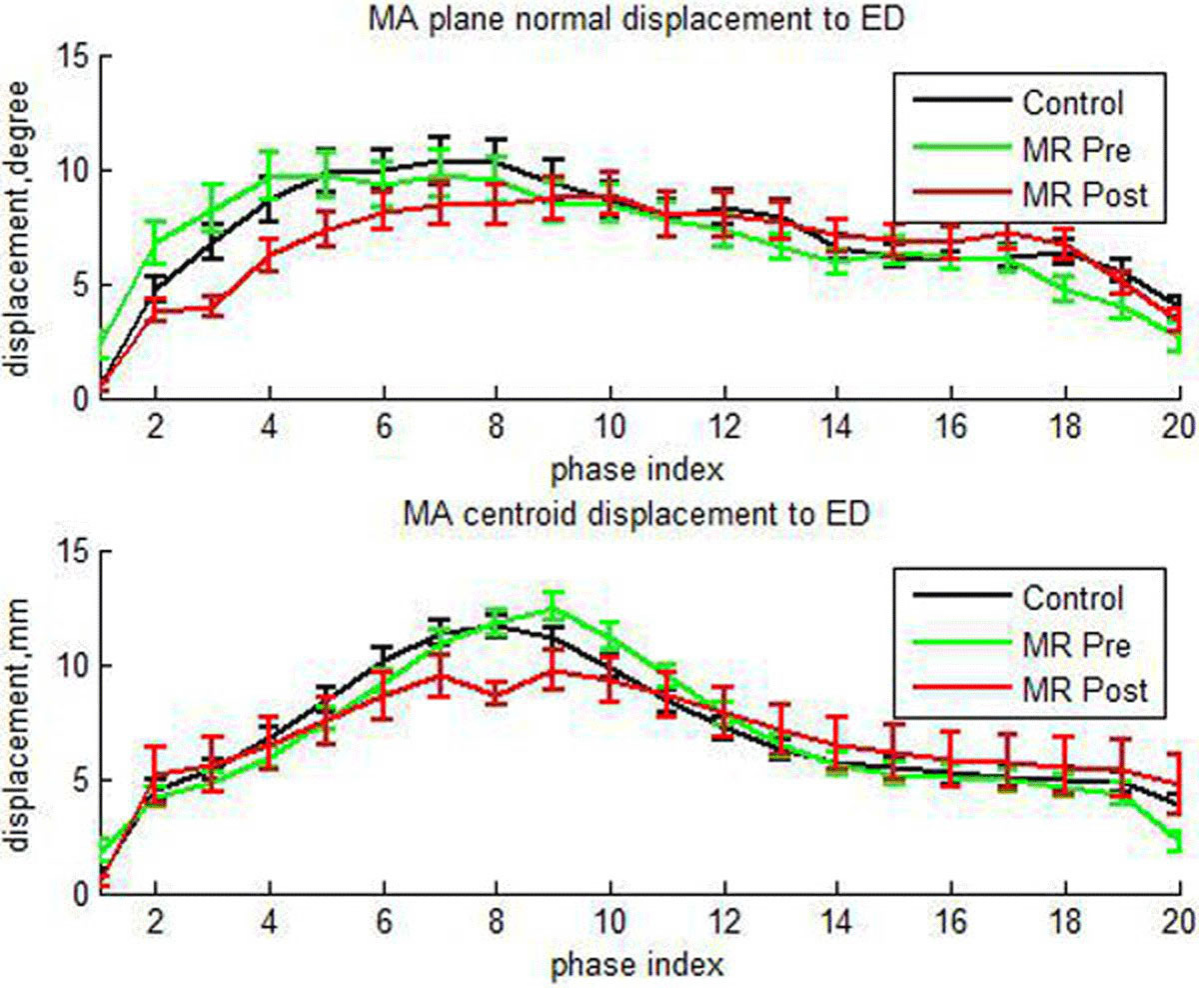
**Mean ± SE MA plane normal and centroid displacement to ED versus phase index**.

Centroid displacement and plane normal angle curves are shown in Figure 1 and parameters of these curves are shown in Table 1.

**Table 1:** MRI-derived MV plane measurements

	Control (n = 51)	MR	
		
		Preoperative (n = 35)	Postoperative (n = 35)
MA Transverse Diameter at ED, mm	33.07 ± 5.52	42.41 ± 7.33*	31.61 ± 3.88†

MA Transverse Diameter at ES, mm	35.95 ± 4.07	45.83 ± 6.68*	32.26 ± 5.1*†

Septolateral Diameter at ED, mm	35.07 ± 3.74	43.73 ± 6.66*	34.51 ± 5.99†

Septolateral Diameter at ES, mm	35.51 ± 3.38	43.58 ± 7.52*	35.01 ± 5.27†

C. to A. distance at ED, mm	95.40 ± 7.75	100.75 ± 8.95*	96.65 ± 9.21

C. to A. distance at ES, mm	79.94 ± 7.49	85.27 ± 8.66*	84.66 ± 8.02*

Left Ventricle Ejection Fraction,%	64 ± 7	61 ± 7*	54 ± 8*†

Peak MA Anterior Displacement, mm	10.53 ± 3.09	11.71 ± 3.71	8.16 ± 2.8*†

Peak E. Dia MA Anterior velocity, % LA length/s	64.02 ± 25.1	76.74 ± 23.63*	55.19 ± 19.64†

Peak MA Inferior Displacement, mm	12.73 ± 3.94	12.85 ± 3.52	8.6 ± 2.43*†

Peak E. Dia MA Inferior velocity, % LA length/s	64.93 ± 35.6	58.82 ± 21.97	39.83 ± 18.47*†

Peak MA Lateral Displacement, mm	13.38 ± 3.23	13.91 ± 3.09	10.39 ± 3.61*†

Peak E. Dia MA Lateral velocity, % LA length/s	79.32 ± 34.3	78.45 ± 24.73	49.4 ± 16.36*†

Peak MA Septal Displacement, mm	10.59 ± 3.55	12.04 ± 3.1	7.67 ± 2.44*†

Peak E. Dia MA Septal velocity, % LA length/s	71.26 ± 27.44	77.18 ± 39.92	43.03 ± 16.9*†

Peak C. to A. dis. Displacement to pre., mm	4.004 ± 1.46	3.759 ± 1.35	2.714 ± 0.85*†

Peak normal displacement to ED, degree	14.65 ± 6.68	14.46 ± 5.53	13.59 ± 5.71

Values are mean ± SE. MR, mitral regurgitation patients; ED: end diastole; ES: end systole; E Dia., early diastole; MA, mitral annulus; C., centroid; A., apex; dis., distance; pre., previous; LA, long axis; *, P < 0.05 vs. Control; †, P < 0.05 vs.Preoperative.

## Conclusions

MR mitral annulus transverse diameter and septolateral diameter were significantly dilated in both ED and ES at baseline vs. controls, but were returned to normal level after surgery. However, we found that mitral annular mechanics in MR patients were impaired at 1 year post-surgery, demonstrated by the significant decrease in peak mitral annulus anterior, inferior, lateral and septal displacements as well as the peak early diastolic mitral annulus velocities normalized to the LVED length vs. controls.

## Funding

NIH/NHLBI P50-HL077100, R01 HL104018.

